# Possibility of Using House Cricket (*Acheta domesticus*) or Mulberry Silkworm (*Bombyx mori*) Pupae Meal to Replace Poultry Meal in Canine Diets Based on Health and Nutrient Digestibility

**DOI:** 10.3390/ani11092680

**Published:** 2021-09-13

**Authors:** Sathita Areerat, Pipatpong Chundang, Chalermpol Lekcharoensuk, Attawit Kovitvadhi

**Affiliations:** 1 Graduate Student in Animal Health and Biomedical Science Program, Faculty of Veterinary Medicine, Kasetsart University, Bangkok 10900, Thailand; sathitameen@gmail.com; 2 Department of Physiology, Faculty of Veterinary Medicine, Kasetsart University, Bangkok 10900, Thailand; pichandang@gmail.com; 3 Department of Companion Animals Clinical Sciences, Faculty of Veterinary Medicine, Kasetsart University, Bangkok 10900, Thailand; fvetcpl@ku.ac.th

**Keywords:** dog, haematology, blood chemistry, apparent digestibility, cricket, silkworm pupae

## Abstract

**Simple Summary:**

Pet foods are one of the fastest-growing products owing to demands by pet owners. In general, soybean meal, meat, meat by-products, meat, and bone meal and fishmeal have usually served as protein sources in canine diets, but they are not sustainable and fluctuate in chemical composition. Recently, several companies around the world have invested in producing edible insects as an alternative protein source for humans, livestock, aquatic animals, and dogs; however, regulation is unique for each country, which has limited the growth of insect industries. Based on several study reports, dogs have been shown to consume diets containing insects without adverse effects on health or nutrient digestibility. House cricket (*Acheta domesticus*: AD) and mulberry silkworm (*Bombyx mori*: BM) pupae are other potential insects able to be good protein sources that could replace poultry meal and fishmeal. Healthy adult mixed-breed dogs were divided into five groups fed diets with 10% AD, 20% AD, 7% BM, or 14% BM for 29 days. During the experiments, dogs were evaluated in terms of blood parameters and nutrient digestibility. The study shown AD and BM, at levels of up to 20% and 14%, respectively, can be a substitute for poultry meal in canine diets without any adverse effects on health and nutrient digestibility.

**Abstract:**

There has been increasing interest in using insects as sustainable protein sources for humans and animals. Therefore, this study aimed to explore the possibility of substituting poultry meal with house cricket (*Acheta domesticus*: AD) or mulberry silkworm (*Bombyx mori*: BM) pupae. Fifty healthy adult mixed-breed dogs were selected and divided into five experimental groups, which were fed, in amounts based on daily energy requirement, with a control diet, a diet with 10% AD, with 20% AD, with 7% BM, or with 14% BM. Days 0–22 and 23–28 of the experiment served as the adaptation and collection phases, respectively. Haematology and blood chemistry were collected at days 0, 14, and 28, and body weight, body condition score, feed intake, faecal output, faecal score, faecal moisture, and apparent total tract digestibility of dry matter and nutrients were measured during the collection phase. The results from this study suggested that AD and BM can replace poultry meal without any adverse consequences on all measured parameters (*p* > 0.05). Therefore, AD at 20% or BM at 14% can be used in canine diet formulations. However, long-term feeding trials should be conducted and should focus on clinical signs relating to hypersensitivity disorders.

## 1. Introduction

As human lifestyle has changed, humans have begun to consider pets not as animals, but rather as family members. Based on this perception, the demand for pet food has increased quickly following this trend. Therefore, it is necessary for pet feed manufacturers to create new products to support them. In general, soybean meal, meat, meat by-products, meat, and bone meal and fishmeal have served as protein sources in canine diets, but they are not sustainable and are of variable chemical composition. Insects are considered as a sustainable alternative protein source and have benefits of a good nutritional profile [1], short production cycle, low investment cost, low area required for production, being environmentally friendly [2], contributing to circular economies [3], and having a higher ratio of edible components than meat or fish [4]. The allowance to use insects as well as insect components as diets for livestock, aquatic species, and companion animals is different between countries, which has made international trading difficult. However, the regulation will be change in the future based on current knowledge on safety after using insects. For example, several states of the United States of America have permitted the use of insects as ingredients in pet food [5]. Therefore, insects have potential as alternative protein sources for animals, particularly dogs. Based on several study reports, dogs have been shown to be able to consume diets containing black soldier fly (*Hermetia illucens*) larvae [6,7,8,9,10,11], tropical house cricket (*Gryllodes sigillatus*) [12], housefly (*Musca domestica*) larvae, lesser mealworm (*Alphitobius diaperinus*) [6], and yellow mealworm (*Tenebrio molitor*) [6,7] without adverse effects on health or nutrient digestibility, even if used as almost total replacements for common protein sources [6]. House cricket (*Acheta domesticus*: AD) and mulberry silkworm (*Bombyx mori*: BM) pupae are other potential insects able to be produced on a large scale. These insects have good nutritional value and digestibility, very similar to poultry meal and fishmeal [6,13]. However, few studies have investigated the use of these insects in canine food. Therefore, this study attempts to show that AD and BM are able to be used as substitutes for poultry meal in canine food by evaluating their health effects and nutrient digestibility.

## 2. Materials and Methods

### 2.1. Animals and Housing

This experiment was conducted at an experimental dog farm (Nakhon Nayok, Thailand). Fifty adult mixed-breed dogs (26 males and 24 females), 2–4 years old with an initial body weight of 26.4 ± 1.22 kg and body condition score of 4.48 ± 0.18 (mean ± Standard error of mean), were selected randomly from the population for this study. All dogs were healthy and passed a physical examination by a veterinarian. Each dog was housed in an individual pen (2 × 2 × 3 m) in an open housing system throughout the experiment to prevent the consumption of foreign material. The experiment was approved by the Institutional Animal Care and Use Committee of Kasetsart University, Bangkok, Thailand (ACKU64-VET-010).

### 2.2. Insects, Diets and Feeding

Frozen AD and BM were purchased from a local company (Pathum Thani, Thailand). All insects were dried at 60 °C for 48 h, ground into 1 mm particles, and kept at −20 °C until use for the experiment. These processes were conducted at the animal nutrition laboratory (Faculty of Veterinary Medicine, Kasetsart University). Dogs were assigned randomly into five experimental groups with 10 dogs per group. Group 1: fed poultry meal as major protein source (CON); Group 2: fed a diet with 10% AD (10% AD); Group 3: fed a diet with 20% AD (20% AD); Group 4: fed a diet with 7% BM (7% BM); and Group 5: fed a diet with 14% BM (14% BM). The insect meal partially replaced poultry meal. For diet preparation, the raw materials were homogenised, clean water was added, and it was mixed until reaching a firm texture. The feed, 7 mm thick, was placed in a stainless-steel tray and baked at 170 °C for 40 min in an electric oven. The baked feed was cooled to room temperature (25 °C), then cut into 2.5 × 2.5 cm pieces, and kept at −20 °C until feeding. The isocaloric and isonitrogenic diets for the studied groups were formulated to comply with the Association of American Feed Control Officials 2021 guidelines for maintenance of adult dogs [14]. The chemical composition of AD and BM were analysed before feed formulation [15] using a nitrogen-to-protein conversion factor of 4.76 [16]. The chemical compositions of experimental diets and feces were proximate analysed in triplicate using a nitrogen-to-protein conversion factor of 6.25 [15]. The ingredients and chemical compositions of the experimental diets are presented in Table 1.

Dogs were fed once per day at 15:00 and water was provided *ad libitum*. The remaining feed was removed at 09:00 on the following day. The feed was removed from the freezer to reach ambient temperature before feeding. Feed amounts were calculated based on the daily energy requirement (DER) of each dog [14]. Days 0–22 and 23–28 were used as adaptation and collection phase, respectively.

### 2.3. Sample and Data Collection

Blood samples (3 mL) were collected from each dog from the left cephalic vein on days 0 (baseline), 14, and 28 of the experimental period. The blood samples were divided into ethylene diamine tetra-acetic acid (EDTA) and plain serum tubes to determine haematology and blood chemistry profiles, respectively. All blood samples were submitted for laboratory analysis at the Veterinary Diagnostic Laboratory Kasetsart University Veterinary Teaching Hospital (Bangkok, Thailand).

Body weight and nine-scale body condition score [17] were measured before beginning the collection phase. Faeces were collected daily at 10:00, weighed, pooled with those of the same dog, homogenised, and stored at −20 °C during the collection phase. Feed intake was also recorded. The chemical components of experimental diets and faeces were analysed [15] to calculate apparent total tract digestibility of dry matter, organic matter, crude protein, and crude fat, following the method of Kilburn et al. [12].

Faecal moisture was evaluated following Association of Official Agricultural Chemists (AOAC) [15], and faecal scores were recorded during the collection phase according to the Purina Faecal Scoring System (Purina Faecal Scoring System for Dogs and Cats, Nestle-Purina Pet Food Co., St. Louis, MO, USA): Score 1, very hard and dry; 2, firm, but not hard; 3, log-like; 4, very moist (soggy); 5, very moist, but with distinct shape; 6, with texture, but no defined shape; and 7, watery, no texture, flat.

### 2.4. Statistical Analysis

A completely randomised design was used in this study. The differences in haematology and blood chemistry between groups were revealed by two-way mixed ANOVA with Duncan’s multiple range test as post-hoc analysis. The groups (between-subject) and collecting sample date (within-subject) served as fixed and random factors, respectively. The results of body weight, feed intake, faecal output, and apparent digestibility were evaluated by one-way ANOVA with Duncan’s multiple range test as post-hoc analysis. Further, body condition score, faecal score, and faecal moisture were evaluated by the Kruskal–Wallis test with Dunnett’s test as post-hoc analysis. The normal distribution and homogeneity of variance were confirmed by Shapiro–Wilk test and Levene’s test, respectively. Statistically significant difference was accepted at *p* < 0.05. All statistical analyses in the study were investigated using R-statistic software (Vienna, Austria) [18] under RStudio ver.1.4.1103 with Rcmdr packages.

## 3. Results

### 3.1. Chemical Compositions of Insect Meals

One hundred grams of AD dry matter contained crude protein (nitrogen-to-protein conversion factor 4.76), crude fat, crude fibre, and ash of 54.4, 16.7, 8.53, and 5.82 g, respectively. The BM dry matter contained crude protein (nitrogen-to-protein conversion factor 4.76), crude fat, crude fibre, and ash of 43.8, 30.1, 3.24, and 4.30 g, respectively.

### 3.2. Haematology and Blood Chemistry

The results of haematology and blood chemistry are presented in Table 2. There were no significant differences between the control and experimental groups (*p* > 0.05) and all parameters were within normal reference values. The mean number of eosinophils of the dogs fed BM was higher compared with other groups, but the difference was not statistically significant (*p* > 0.05).

### 3.3. Body Weight, Body Condition Score, Feed Intake, Faecal Output, Faecal Score, Faecal Moisture, and Apparent Total Tract Digestibility

There were no significant differences between groups in body weight, body condition score, feed intake, faecal output, faecal score, and faecal moisture or apparent total tract digestibility of dry matter, organic matter, crude protein, and crude fat (Table 3). The average faecal score of dogs fed BM was higher than other groups, but no statistically significant difference was observed (*p* = 0.05).

## 4. Discussion

### 4.1. Diets and Insects

AD and BM can be considered as alternative protein sources in canine nutrition. The chemical composition of insects is close to that of common protein sources [19]. In this study, all diets were formulated as isocaloric and isonitrogenous based on calculation. The higher analysed crude protein value can be explained by the differences in the nitrogen-to-protein conversion factor; that used for insects during formulation was 4.76 [16], whereas for other raw materials and experimental diets, 6.25 was used [15]. The total nitrogen contents of each ingredient—soybean meal, corn, wheat flour, poultry meal, house cricket meal and silkworm pupae meal—were 8.09, 1.36, 2.30, 11.1, 11.2, and 9.02, respectively. Consequently, the calculated crude protein from isonitrogenous diets using insect meals resulted in a lower value than for analysed crude protein. The greater analysed crude protein level based on the Kjeldahl method is because insect skeletons contain a modified polysaccharide containing nitrogen called chitin [16]. Therefore, the use of a lower value of nitrogen-to-protein conversion factor during the formulation of insect meals results in the true protein level of experimental diets being nearly equal. In contrast, using 6.25 as the nitrogen-to-protein conversion factor for insect meals could result in the true protein level in a diet using insect meal being lower than the control diet using common protein sources, and affect the measuring parameters. However, the digestible crude protein and metabolisable energy of insect meals could be analysed in dogs and achieve high accuracy in feed formulation. Based on our study, crude fibre was increased following the amount of insect inclusion, which was same as in other studies [12,20]. Chitin could be the major factor influencing this consequence.

### 4.2. Health Parameters

Haematology and blood chemistry can indicate an animal’s health and are generally used in veterinary medicine. General canine health is not affected after ingestion of diets containing defatted black soldier fly larvae meal [8] or tropical house cricket [12] based on blood profiles. A linear increase in serum albumin and calcium levels with respect to the supplementation level of defatted black soldier fly larvae meal in dog feed has been reported [8]. Moreover, slight increases in blood urea nitrogen and haemoglobin were found in dogs fed a diet containing tropical house cricket [12]. The higher protein intake induced higher serum albumin and blood urea nitrogen as their metabolites. Higher crude protein levels in diets were presented in the study by Lei et al. [8] and Kilburn et al. [12], which could be the cause of the increases in these blood parameters. On the one hand, the albumin and blood urea nitrogen seem to increase in dogs fed insect meals compared with the control group in this study, even if isonitrogenous diets were used and feed intake was not different between groups. However, there was no statistically significant difference in these parameters (*p* > 0.05). Therefore, these blood parameters could be not affected by the addition of insects. Based on this result, eosinophil levels seem to be higher in all insect-addition groups compared with the control group, but with no statistically significant difference (*p* = 0.10), whereas this trend was not observed in other studies [8,12]. Food allergies can promote eosinophilia [21] and insects are suspected as allergens. Premrov Bajuk et al. [22] suggested that mite allergies in dogs may involve cross-reactivity with yellow mealworm larvae proteins. The problems of allergenic reactions in dogs fed an insect-based diet need more evidence to clarify. However, no allergenic signs were observed in this study. Moreover, all blood parameters were in the normal range with no statistical or biological significance. Therefore, feeding dogs with AD or BM did not result in a deterioration in blood parameters.

In the adaptation period, each dog was adjusted to an ideal body condition score of 4–5. There were no significant differences in body weight, body condition score, feed intake, or faecal output in the experiment group (*p* > 0.05), findings matching those observed in earlier studies [12]. However, a higher faecal output was seen after feeding tropical house cricket because the indigestible content, chitin, is higher in crickets compared with chicken meal [12]. This study did not find differences in this parameter between groups. The diverse chemical composition and digestibility of insect meals could be the cause of this difference.

The faecal score is an easy tool to determine the acceptance and digestibility of food, in which the ideal faecal quality must be firm to soft and maintain its shape [19]. In this study, there were no differences in faecal score or the ideal stool quality, the same outcomes as seen in other studies using black soldier fly larvae [10] or tropical house cricket meal [12] in dog diets. The faecal score seemed to be high in the 7% BM and 14% BM groups, with no statistically significant difference between groups, all showing an ideal score. Therefore, AD or MB can be fed to dogs without adverse consequences on the faecal score.

### 4.3. Total Tract Apparent Digestibility

The digestibility of dry matter, organic matter, and fat is not influenced by the use of black soldier fly larvae [7,8,9,10,11], yellow mealworm [7], or tropical house cricket [12] in canine feed, which agreed with this study. Therefore, dogs can be fed these insects without problems in these digestibility parameters. Digestibility of crude protein is one of indicators that reveals the quality of the protein source. In general, good quality protein sources have a digestibility of around 80% in dogs [23]. The in vitro digestibility and fermentation of crude house cricket protein is close to those of poultry meal and soybean meal in dogs [6]. The apparent total tract digestibility of crude protein by dogs consuming diets containing black soldier fly larvae [7,8,9,10,11], yellow mealworm [7], or tropical house cricket [12] was 77.1–87.2%, 83.6%, and 82.1–86.0%, respectively. However, the apparent crude protein digestibilities of AD (75.4–76.9%) and BM (77.3–78.0%) in this study were lower than seen in other studies, but similar to the control diets (72.8%). Differences in protein source (poultry meal, venison, or lamb), raw material quality, formulation, and animals could be an important factor influencing the different values of crude protein digestibility. Moreover, different crude protein proportions in the control diet were set at 18.1–32.0%DM in other studies [8,9,11,12,20], whereas in this study, the crude protein content was 23.5%DM. Therefore, from the crude protein digestibility lower than 80% found in this study, one still cannot conclude that AD and BM are not a good quality protein source because the crude protein digestibility was similar to the control diet using poultry meal. The value of digestible protein in insect meals for dogs could be beneficial in feed formulation. Therefore, further work is required to reveal the crude protein digestibility for these insects compared to common protein raw materials.

## 5. Conclusions

The purpose of the current study was to determine the possibility of using house cricket (*Acheta domesticus*: AD) or mulberry silkworm pupae (*Bombyx mori*: BM) in canine diets by evaluating blood parameters and nutrient digestibility. The research has shown that AD and BM, at levels of up to 20% and 14%, respectively, can substitute for poultry meal in canine diets without adverse effects on haematology, blood chemistry, body weight, body condition score, feed intake, faecal output, faecal score, faecal moisture, or apparent total tract digestibility (*p* > 0.05). A further study could assess the long-term effects of the ingestion of insect-based diets, particularly with regard to hypersensitivity disorders.

## Figures and Tables

**Table 1:** Ingredients and chemical compositions of the experimental diets.

Items	House Cricket Meal	Silkworm Pupae Meal	Groups					AAFCO ^1^
			Control	House Cricket Inclusion		Silkworm Pupae Inclusion		
				**10%**	**20%**	**7%**	**14%**	
Ingredients (%)								
Corn	-	-	50.1	48.1	46.2	48.7	47.2	-
Soybean meal	-	-	10.0	10.0	10.0	10.0	10.0	-
Wheat flour	-	-	15.0	15.0	15.0	15.0	15.0	-
Poultry meal	-	-	17.4	10.0	2.51	13.3	9.10	-
House cricket meal	-	-	-	10.0	20.0	-	-	-
Mulberry silkworm pupae meal	-	-	-	-	-	7.00	14.0	-
Palm oil	-	-	5.72	5.09	4.44	4.01	2.50	-
Calcium carbonate	-	-	0.53	0.56	0.60	0.74	0.95	-
Salt	-	-	0.60	0.60	0.60	0.60	0.60	-
Vitamin premix ^2^	-	-	0.15	0.15	0.15	0.15	0.15	-
Mineral premix ^3^	-	-	0.20	0.20	0.20	0.20	0.20	-
Choline chloride	-	-	0.30	0.30	0.30	0.30	0.30	-
Calculated composition (%DM)								
Metabolizable energy (kcal/kg) ^4^	-	-	3700	3700	3700	3700	3700	-
Crude protein ^5^	-	-	23.5 (23.5)	23.5 (25.3)	23.5 (26.9)	23.5 (24.5)	23.5 (25.4)	18.0
Crude fat	-	-	10.3	10.3	10.3	10.2	10.1	5.50
NFE ^6^	-	-	57.2	57.1	57.1	57.4	57.6	-
Crude fibre	-	-	2.67	3.30	3.93	2.76	2.85	-
Ash	-	-	6.03	5.32	4.61	5.77	5.52	-
Methionine	-	-	0.39	0.38	0.37	0.43	0.46	0.33
Cystein	-	-	0.32	0.29	0.27	0.31	0.31	-
Methionine + cystein	-	-	0.71	0.67	0.65	0.74	0.76	0.65
Lysine	-	-	1.13	1.05	0.96	1.14	1.16	0.63
Calcium (Ca)	-	-	0.94	0.78	0.62	0.87	0.80	0.50
Phosphorus (P)	-	-	0.75	0.62	0.49	0.69	0.64	0.40
Ca/P ratio	-	-	1.25	1.25	1.25	1.25	1.25	1.00–2.00
Analyzed composition (%DM)								
Dry matter	94.1	94.0	79.3	78.8	70.5	84.7	77.1	-
Crude protein ^7^	54.4	43.8	25.3	26.4	29.2	25.0	25.7	18.0
Crude fat	16.7	30.1	10.5	10.3	10.2	10.0	10.4	5.50
Crude fibre	8.53	3.24	1.89	2.45	3.97	2.02	2.30	-
Ash	5.82	4.30	5.16	4.08	3.78	4.78	4.79	-

^1^ Association of American Feed Control Officials 2021 dog food nutrient profiles based on dry matter recommendations for adult maintenance [14]. ^2^ Vitamin premix (Feed specialties Co., Ltd.; Pathumthani, Thailand) was supplied per kilogram of diets at 2,500,000 IU of vitamin A; 1,000,000 IU of vitamin D3; 7000 IU of vitamin E; 700 mg of vitamin K; 400 mg of vitamin B1; 800 mg of vitamin B2; 400 mg of vitamin B6; 4 mg of vitamin B12; 30 mg of biotin; 3111 mg of Ca pantothenate acid; 100 mg of folic acid; 15,000 mg of vitamin C; 5600 mg of vitamin B3. ^3^ Mineral premix (Feed specialties Co., Ltd.; Pathumthani, Thailand) was supplied per kilogram of diets at 10,500 mg of Zn, 10,920 mg of Fe; 9960 mg of Mn; 3850 mg of Cu; 137 mg of I; 70 mg of Se. ^4^ Modified Atwater values: Metabolizable energy (kcal/kg) = (Protein × 3.5) + (Fat × 8.5) + (Carbohydrate × 3.5). ^5^ Nitrogen-to-protein conversion factor at 4.76 or 6.25 was used for the insect meal and other ingredients in feed formulation, respectively [16]. The number in the blanket represented the crude protein, which was calculated from the nitrogen-to-protein conversion factor at 6.25 for all ingredients. ^6^ Nitrogen-free extract. ^7^ Nitrogen-to-protein conversion factor for analyzed composition was used at 6.25.

**Table 2:** Haematology and blood chemistry in mixed-breed dogs (2–4 years old; n = 10 per group) with or without the inclusion of house cricket (*Acheta domesticus*) or mulberry silkworm (*Bombyx mori*) pupae in diets.

Parameters ^1^	Groups					SEM	*p*-Value	Reference Value ^2^
	Control	House Cricket Inclusion		Silkworm Pupae Inclusion				
		**10%**	**20%**	**7%**	**14%**			
Haemoglobin (g/dL)	17.7	15.3	16.7	16.7	16.5	0.247	0.40	13.1–20.5
Haematocrit (%)	47.5	42.2	45.2	45.2	43.9	0.979	0.17	37.3–61.7
Red blood cell (10^6^/uL)	8.07	6.99	7.24	7.93	7.43	0.101	0.09	5.65–8.87
MCV (fL)	58.9	60.2	62.4	56.9	59.3	1.031	0.11	61.6–73.5
MCHC (g/dL)	37.7	36.8	37.4	37.3	37.8	0.685	0.99	32.0–37.9
MCH (pg)	22.0	21.9	23.0	21.0	22.2	0.148	0.38	21.2–25.9
White blood cell (10^3^/uL)	9.44	15.7	12.8	12.0	13.9	1.724	0.32	5.05–16.8
Neutrophils (10^3^/uL)	5.85	9.83	8.11	5.96	8.91	0.883	0.06	2.95–11.6
Lymphocytes (10^3^/uL)	2.50	2.87	3.11	3.64	2.64	0.603	0.68	1.05–5.10
Monocytes (10^3^/uL)	0.47	1.18	0.54	0.60	0.70	0.254	0.44	0.16–1.12
Eosinophils (10^3^/uL)	0.61	1.05	1.02	1.79	1.60	0.287	0.10	0.06–1.23
Basophils (10^3^/uL)	0.02	0.05	0.02	0.03	0.04	0.007	0.11	0.00–0.10
Platelets (10^3^/uL)	299	278	275	237	245	11.22	0.51	148–484
RDW (fL)	19.0	16.7	18.0	18.3	17.3	0.307	0.35	9.10–19.4
MPV (fL)	10.4	12.0	12.4	11.3	11.9	0.132	0.06	8.70–13.2
Blood urea nitrogen (mg%)	12.5	13.1	13.4	13.8	13.6	0.480	0.99	10.0–26.0
Creatinine (mg%)	1.07	0.96	1.22	1.20	1.15	0.024	0.41	0.50–1.30
ALT (IU/L)	47.3	29.3	34.0	28.3	35.1	2.897	0.66	6.00–70.0
Total protein (gm%)	7.20	7.16	6.36	6.73	6.87	0.089	0.47	5.30–7.80
Albumin (gm%)	2.98	3.01	3.22	3.20	3.02	0.038	0.59	2.30–3.20
Globulin (gm%)	4.23	4.14	3.13	3.53	3.84	0.101	0.35	2.70–4.40

^1^ MCV; mean corpuscular volume, MCHC; mean corpuscular hemoglobin concentration, MCH; mean corpuscular hemoglobin, RDW; red cell distribution width, MPV; mean platelet volume ALT; alanine aminotransferase. ^2^ Reference intervals were derived from the Veterinary Diagnostic Laboratory Kasetsart University Veterinary Teaching Hospital, Bangkok, Thailand.

**Table 3:** Body weight, body condition score, feed intake, faecal output, faecal score, faecal moisture, and apparent total tract digestibility (between day 23 and 28) in mixed-breed dogs (2–4 years old; n = 10 per group) with or without inclusion of house cricket (*Acheta domesticus*) or mulberry silkworm (*Bombyx mori*) pupae in diets.

Parameters	Groups					SEM	*p*-Value
	Control	House Cricket Inclusion		Silkworm Pupae Inclusion			
		**10%**	**20%**	**7%**	**14%**		
Body weight at d 22 ^1^	27.3	24.1	24.8	25.7	26.4	2.686	0.86
Body condition score at d 22	4.73	4.27	4.57	4.23	4.47	0.384	0.91
Intake							
Feed intake, g as fed/d	371	400	453	381	434	17.25	0.54
Feed intake, g DM/d	468	508	642	450	564	24.37	0.06
Output							
Fecal output, g as-is/d	247	213	234	187	266	12.39	0.32
Fecal output, g of DM/d	94.8	87.4	92.2	70.8	95.3	4.426	0.37
Fecal score	3.13	3.11	3.30	3.78	3.57	0.176	0.59
Fecal moisture (%FM)	61.0	61.3	61.9	61.0	63.8	0.560	0.38
Apparent total tract digestibility (%)							
Dry matter	71.5	75.2	74.8	79.7	74.8	1.002	0.19
Organic matter	73.6	76.9	76.0	79.9	77.9	0.947	0.40
Crude protein	72.8	75.4	76.9	78.0	77.3	0.924	0.50
Crude fat	93.8	92.7	92.0	95.1	92.8	0.527	0.38

^1.^ d 22; the day before the collection phase after the dog’s body weight and body condition score were adjusted.
